# Local Pulmonary Immunological Biomarkers in Tuberculosis

**DOI:** 10.3389/fimmu.2021.640916

**Published:** 2021-03-05

**Authors:** Hazel Morrison, Helen McShane

**Affiliations:** The Jenner Institute, University of Oxford, Oxford, United Kingdom

**Keywords:** tuberculosis, biomarkers, pulmonary, mucosal, vaccines

## Abstract

Regardless of the eventual site of disease, the point of entry for *Mycobacterium tuberculosis (M.tb)* is via the respiratory tract and tuberculosis (TB) remains primarily a disease of the lungs. Immunological biomarkers detected from the respiratory compartment may be of particular interest in understanding the complex immune response to *M.tb* infection and may more accurately reflect disease activity than those seen in peripheral samples. Studies in humans and a variety of animal models have shown that biomarkers detected in response to mycobacterial challenge are highly localized, with signals seen in respiratory samples that are absent from the peripheral blood. Increased understanding of the role of pulmonary specific biomarkers may prove particularly valuable in the field of TB vaccines. Here, development of vaccine candidates is hampered by the lack of defined correlates of protection (COPs). Assessing vaccine immunogenicity in humans has primarily focussed on detecting these potential markers of protection in peripheral blood. However, further understanding of the importance of local pulmonary immune responses suggests alternative approaches may be necessary. For example, non-circulating tissue resident memory T cells (T_RM_) play a key role in host mycobacterial defenses and detecting their associated biomarkers can only be achieved by interrogating respiratory samples such as bronchoalveolar lavage fluid or tissue biopsies. Here, we review what is known about pulmonary specific immunological biomarkers and discuss potential applications and further research needs.

## Introduction

Tuberculosis (TB) remains one of the top ten causes of death worldwide. Around a quarter of the world's population are estimated to be infected with *Mycobacterium tuberculosis* (*M.tb*) (1). The World Health Organization's (WHO) End TB Strategy has set the goals of reducing TB incidence by 90% and TB deaths by 95% globally by 2035. If there is any chance of meeting these ambitious targets, new tools to combat this devastating disease will be needed. These include an urgent need for improved diagnostic tests, shorter treatment regimens and more effective vaccines (2).

The range of clinical phenotypes following *M.tb* exposure spans complete elimination of the pathogen through immunologically contained latent infection to active TB disease (3). This spectrum is governed by complex and incompletely understood interactions between the pathogen and host innate and adaptive immune responses. Innate immune mechanisms within the lung mucosa may be responsible for early clearance of *M.tb* bacilli prior to T-cell sensitization in exposed individuals who appear to be resistant to *M.tb* infection (4). Of those who do have presumed latent *M.tb* infection (LTBI), 5–10% of immunocompetent individuals go on to develop TB disease in their lifetime (5), with the remaining majority achieving immunological equipoise.

Incomplete knowledge of the desired immune responses needed to prevent either active disease or initial infection is one of the key barriers to effective vaccine development (6). It is well-characterized that a T-helper 1 (Th1) cell-mediated adaptive immune response is required, but insufficient, for protection (7, 8). Likewise, whilst antigen-specific interferon gamma (IFN-γ) plays a key role, the level of vaccine-induced IFN-γ in the blood does not correlate with protection (9, 10). Understanding the host immune responses that are needed to confer adequate protection against *M.tb* would dramatically help in the development and prioritization of vaccines that induce these putative responses.

An immunological biomarker is a measurable characteristic of the immune system that can be assessed as an indicator of normal immune function, disease process, or response to a therapeutic intervention (11). Biomarkers of disease can be used in diagnosis and disease monitoring. Vaccines aim to induce an immunological response to prevent infection or reduce disease severity, termed protection. Biomarkers that are believed to correspond with this effect are termed immune correlates of protection (COP) (12) and form the main focus of biomarkers discussed in this review.

The majority of TB studies looking at biomarkers of protection, both from disease and from infection, have focussed on the peripheral blood compartment in humans and blood and lymphoid organs in animal models. Regardless of the site of active disease, the predominant route via which *M.tb* bacilli enter the body is via aerosol droplets that are deposited onto alveolar surfaces of the lungs (4). Systemic immunity does not necessarily reflect pulmonary immune responses in the bronchoalveolar spaces at this site of entry for *M.tb* in humans. Cells of both the innate (such as alveolar macrophages) and adaptive (such as tissue resident memory cells) components of the pulmonary immune system play an increasing recognized role that may be interrogated in the search for markers of protection.

## Innate Immunity Within the Lung

### Trained Immunity

Trained immunity refers to immunological memory within the innate immune system, leading to an augmented response to subsequent, often heterologous insults (13). Innate immune memory is induced in animals after vaccination with BCG (14, 15) although the precise mechanisms via which this occurs are still being studied. Studies of TB contacts show that despite high levels of exposure, up to 30–50% of individuals do not become infected with *M.tb*, as evidenced by non-reactive tuberculin skin tests and negative IFN-γ release assay (IGRA) testing (16). BCG vaccination correlates with this state of immune protection, suggesting that BCG-potentiated innate immunity may contribute to early *M.tb* clearance (17).

Given this, it is unclear why, in mice, BCG does not protect against *M.tb* in the first 14 days post-challenge (18). The kinetics and role of the innate immune response need further study. Controlled human infection models with serial mucosal and systemic sampling allow us to define the kinetics of innate and adaptive immunity and may help us understand this further.

### Alveolar Macrophages

In CD4/CD8 T-cell knock out mice, subcutaneous BCG vaccination induces lasting protective immunity within 7 days, prior to any adaptive immune mechanisms (18). Cells from the lungs of vaccinated mice show a higher proportion of tissue resident macrophages (CD11b^+^F4/80^+^) compared to circulating monocytes. Following an infection, or in this case immunization, monocytes may differentiate into interstitial lung macrophages, which then self-perpetuate within the pulmonary compartment. This may represent a mechanism via which BCG induces innate immune memory within the lung (18).

Respiratory viral infection has been found to induce immune memory in lung resident mouse alveolar macrophages (AMs), which go on to produce accelerated levels of detectable neutrophil chemokines, such as CXCL1 and CXCL2 upon restimulation. These trained AMs protect against secondary bacterial infection, with a memory response that is not reliant on circulating monocytes (19).

In a recent model using T-cell depleted mice, mucosal, but not intramuscular, vaccination with an adenoviral-vectored vaccine expressing the *M.tb* antigen 85A resulted in upregulation of activation markers, such as MHC II, on alveolar and pulmonary interstitial macrophages. This corresponded with reduced *M.tb* burden after challenge and suggests that activated airway macrophages may play an important role in early *M.tb* control (20). Debate is ongoing about the precise role AM play in *M.tb* control. AMs do not readily express pro-inflammatory genes until 10 days after host *M.tb* infection, which may allow early mycobacterial replication (21). AM-depleted mice show defective granuloma formation, but increased recruitment of other phagocytic and cytotoxic cells to the lungs, with corresponding improved *M.tb* clearance (22).

AM have been shown to leave the alveolar space and transport *M.tb* to the lung interstitium in an IL-1 dependent manner, proliferating within the lung to form aggregates (23). Whether this represents the initiation of effective immunological control or the first step in *M.tb* dissemination is not clear. Systemic BCG immunization in mice has been shown to hasten this egress of *M.tb*-infected AM from the alveoli into the lung interstititum, increase attraction of monocyte-derived macrophages to the site of infection and promote the early transfer of *M.tb* from AM to other phagocytic cells (24). In humans, infant AM are less able to control *M.tb* replication *in vitro* than adult AM, which may partly explain their susceptibility to more severe, disseminated forms of TB disease. Infant AM were found to express lower levels of chemotactic cytokines including chemokine (C-X-C motif) ligand 9 (CXCL9), suggesting that failure of AM to recruit additional mononuclear cells to the site of infection may result in failure of initial *M.tb* control (25).

### Innate Lymphoid Cells

Innate lymphoid cells (ILCS) mediate protective immunity in a variety of tissues, including the lungs. Activated ILCs proliferate in the lungs of mice following mucosal BCG vaccination and lead to increased levels of IFN-γ production (26).

Group 3 ILCs (ILC3s) have similar functionality to Th-17 cells, including production of Il-17 and Il-22. In a human lung tissue explant model, ILC3s upregulate IL-22 and GM-CSF following *ex vivo M.tb* infection (27) and IL-22 producing ILCs have been shown to enhance phagolysosomal fusion leading to mycobacterial growth inhibition (28). Inhibition of phagolysosomal fusion is one of the key immune mechanisms whereby *M.tb* evades host immunity.

ILC3s proliferate in the lungs of *M.tb* infected mice, leading to early alveolar macrophage accumulation. ILC knockout mice showed loss of early AM-mediated *M.tb* control, which could be rescued by adoptive cell transfer (ACT) of lung ILCs from *M.tb*-infected control mice (29). ACT of ILC3s also prolonged the survival of diabetic *M.tb*-infected mice, with increased IL-22 production resulting in reduced lung epithelial damage (30). Loss of ILCs, in particular ILC3, leads to a decrease in AM recruitment within the lung and subsequent higher mycobacterial burden during *M.tb* infection (29).

Distinct populations of CD103-expressing ILC2 and ILC3s and CXCR5-expressing ILC3s have been identified in human *M.tb*-infected lung tissue (29). CXCR5 signaling is essential in the formation of inducible bronchus associated lymphoid tissue (iBALT). iBALT is seen surrounding granuloma formation in non-human primate (NHP) and humans with LTBI, but not TB disease (31). iBALT proliferation in the lungs of mice lacking lymph nodes and spleen may be sufficient to control *M.tb* infection (32).

These studies suggest that ILC3s in particular may have a protective role in early *M.tb* control, via CXCR5-dependant iBALT formation and the production of IL-22 and IL-17. Mouse models of intranasal BCG vaccination have shown a correlation between protection and levels of IL-17 producing cells within the lungs following *M.tb* challenge (33).

### Mucosal-Associated Invariant and γδ T Cells

Mucosal-associated invariant T (MAIT) cells preferentially reside in mucosal tissues, including the pulmonary mucosa. They express pattern recognition receptors, conferring innate immune function, and secrete IFN-γ following stimulation. In humans and NHPs, MAIT cells are enriched in the lungs and BAL fluid following *M.tb* infection and NHP MAITs express activation markers such as CD69 following both *M.tb* challenge and intradermal (ID) BCG vaccination (34, 35). In rhesus macaques, intravenous (IV) BCG vaccination induces pulmonary MAIT expansion, which corresponds with subsequent protection against *M.tb* challenge (36). Following *M.bovis* infection, MAIT cell deficient mice show higher bacterial colony forming units (CFUs) at early time points compared to wild-type mice (37), highlighting a potential role for MAITs in early mycobacterial clearance.

γδ T-cells are defined by heterodimeric T-cell receptors (TCRs) composed of γ and δ chains and are enriched in epithelial and mucosal tissues, including lung alveoli. The majority are activated in an MHC-independent manner and produce cytotoxic granules and canonical pro-inflammatory cytokines, including IFN-γ, TNF-α, and IL-17. Their activation results in killing of *M.tb* infected macrophages (38). Following bacterial infection, lung γδ T-cells in mice exhibit increased expression of activation markers such as CD69 and CD25, and proliferate by local expansion rather than recruitment from the periphery (39). In NHPs, expansion of lung γδ T-cells by selective vaccination reduces disease pathology and dissemination following *M.tb* challenge (40).

## Adaptive Pulmonary Immunity

### Lung Tissue Resident Memory Cells

Tissue resident memory cells (T_RM_) represent a distinct subset of lymphocytes. They share functional similarities with central and effector memory T-cells, but remain situated within localized tissue compartments and do not recirculate into the blood stream. They have been demonstrated at sites including the skin, intestines, urogenital tract, and lung mucosa (41–44). This positioning at key anatomical barrier sites means that T_RM_ can respond rapidly to potential infective stimuli and lung T_RM_ may signify the first line of adaptive cellular defense against specific respiratory pathogens, including *M.tb*.

Due to the highly vascular nature of the lungs, distinguishing genuine T_RM_, truly resident in the lung mucosa, from blood lymphocytes that egress from the vasculature following a stimulus such as infection, is difficult. Mouse models, using techniques such as parabiosis and *in vivo* intravascular staining, have confirmed that true lung T_RM_ cells are identifiable and do not re-enter the peripheral circulation, in comparison to lymphoid memory T cells.

Many of the techniques employed in animal models to delineate T_RM_ from pulmonary vascular lymphocytes are not feasible in humans but have been crucial in confirming that biomarkers seen in humans correspond to T_RM_ specific markers identified in animals. Upregulation of CD69 is a key marker of T_RM_ activation at a variety of sites including the lung and results in inhibition of sphingosine 1-phosphate-meditated lymphocyte migration (45). Additionally, CD8^+^ T_RM_ cells express the αEβ7 integrin heterodimer, identified by CD103 marker staining (46). Other significant markers of lung T_RM_ in both human and animal models include PD-1, CD44, CXCR3, and integrins including CD49a, CD11a, and VLA-4 (45), with KLRG-1 and CD62L downregulated (47). CD4^+^ T_RM_ form a heterogeneous group, with some displaying an effector profile (T-bet^+^) and others appearing more regulatory (Foxp3^hi^ IL-10^hi^]. In contrast, pulmonary CD8+ T_RM_ cells appear more homogenous, expressing predominantly Th1 cytokines (48).

The key importance of these cells in animal models of respiratory infection has been shown in several studies. In murine adoptive transfer studies, CXCR3^hi^CD4^+^ T-cells preferentially localize to the lung parenchyma and are better at controlling *M.tb* infection than their CX3CR1^h^iKLRG1^hi^ equivalents which remain within the vasculature (47). Intranasal immunization of mice with a recombinant influenza A vaccine expressing the PR8.p25 Ag85B epitope led to the development CD4^+^ T_RM_ throughout the lung parenchyma. Persistence of these cells following FTY720-induced intravascular lymphopaenia indicates true tissue-resident memory status, without reliance on circulating cells, and was sufficient for protection against subsequent *M.tb* challenge (49).

Route of vaccination may alter the magnitude and character of the adaptive pulmonary immune response, but it is unclear if this will necessarily lead to improved overall protective efficacy. For example, airway mucosal boosting following parental priming with the subunit vaccine candidate H56:CAF01 results in a significant increase in pulmonary T_RM_ and early local T-cell responses, without conferring any additional protection against *M.tb* challenge (50). Intramuscular vaccination of mice with the adjuvanted subunit TB vaccine candidate ID-93 results in a systemic, TH1-dominated immune response. In contrast, following ID-93 intranasal immunization, a predominantly IL-17A-producing, TH-17 response is seen; with an increase in antigen specific CD4^+^ T_RM_ in the lung and BAL fluid. Despite these differences, the level of protection conferred was equal across the different delivery methods (51). In a recent study, protection conferred by intra-tracheal administration of the fusion protein TB vaccine candidate, CysVac2, was associated with the induction of higher levels of antigen-specific CD4^+^ lung T_RM_, expressing IL-17, and RORγT (52).

While intradermal BCG vaccination is able to generate antigen*-*specific pulmonary T_RM_ in mice, mucosal BCG vaccination produces increased numbers of both CD4^+^ and CD8^+^ T_RM_ and this corresponds with subsequent enhanced protection against *M.tb* challenge (48, 53). Mucosal transfer of sorted airway resident T-cells, in particular CD8^+^ T_RM_, from mucosally BCG-vaccinated mice provided increased protection against *M.tb* challenge in recipient mice (48). Non-human primates immunized with intravenous BCG were found to have significantly higher levels of CD69^+^ (with a subset of CD103^+^) lung parenchymal CD4^+^ T-cells than intradermal or aerosol immunized animals and this was associated with sterilizing immunity against *M.tb* challenge (36).

These findings suggest that vaccination routes and strategies which induce pulmonary CD4+ and CD8+ T_RM_ may result in superior levels of protection. This may be one reason why levels of peripheral circulating antigen-specific T-cells do not adequately correlate with protection. Biomarkers of T_RM_ may be useful as correlates of vaccine induced protection, but would require a significant change in sampling methods to assess vaccine efficacy.

### Lung Mucosal Antibodies IgA

The role of the humoral immune system in TB control is uncertain. In humans, *M.tb* infection induces *M.tb*-specific IgA, as well as IgG, antibodies in BAL fluid, but their precise role and level of interaction with *M.tb* at the mucosal level remains unknown (54, 55).

Secretory Immunoglobulin A (sIgA) is the predominant isotype in mucosal secretions and may contribute to protection. Intranasal administration of purified human sIgA to mice leads to increased *M.tb* clearance and improved disease control (56). Knockout mice lacking the polymeric IgR receptor necessary for IgA transport to the respiratory mucosa are more susceptible to *M.tb* infection than wild-type mice (57). In a BCG challenge model, IgA deficient mice are more susceptible to infection than wild-type (58).

## Linking the Innate and Adaptive Immune System

A functional mycobacterial growth inhibition assay (MGIA), which measures the sum of the parts of the innate and adaptive immune response, may be a useful tool to facilitate vaccine development. Such a tool could also allow the interrogation of potential COP by depletion studies using serum, peripheral blood mononuclear cells (PBMCs) or other specific cell types. To date such an assay has been optimized for use in whole blood and PBMC (59, 60). Using mucosal samples in such an assay may further identify lung specific protective mechanisms in future.

## Interrogating Pulmonary Mucosal Immunity

Animal and human studies that focus on sampling the lung mucosal compartment will improve our understanding of lung mucosal immunity to *M.tb*. Parallel animal and human studies would allow more detailed interrogation of these processes. Delivery of vaccine candidates via aerosol routes has been shown to induce specific mucosal immune components that can be compared across species, with BAL samples from macaques and humans following aerosol MVA85A showing increased levels of antigen-specific cellular immune responses compared to peripheral blood (61–63). Further detailed mechanistic interrogation of lung-specific immunity is possible in the more tractable murine model (64, 65).

### Specific Challenges of Human Studies

**Figure 1:**
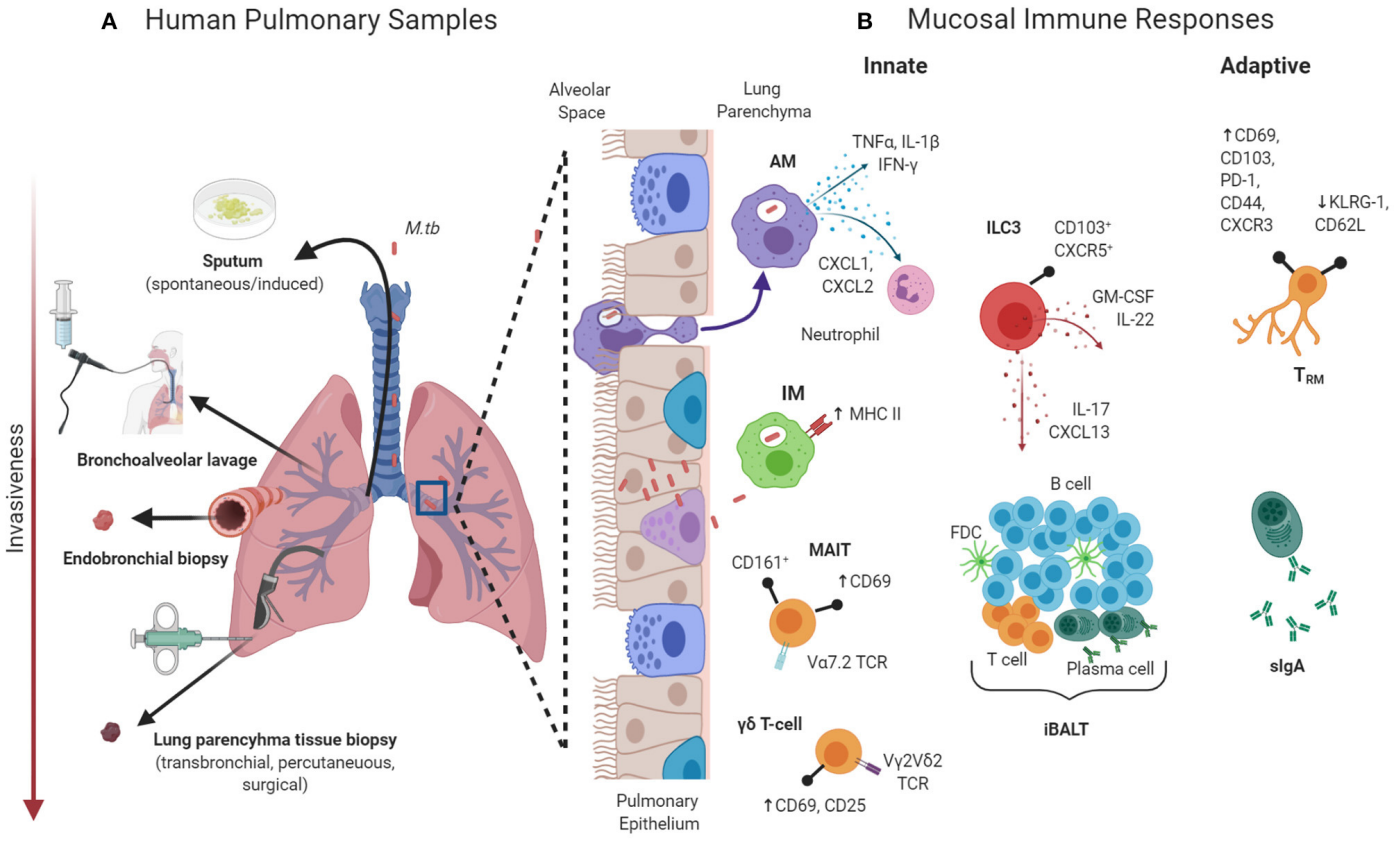
**(A)** Samples that can be obtained from the human pulmonary compartment from immunological interrogation. **(B)** Selected components of the pulmonary mucosal immune system that may be involved in protection against *Mycobacterium tuberculosis (M.tb)*. AM, alveolar macrophages; CD, cluster of differentiation; CXCR, C-X-C chemokine receptor; FBC, follicular dendritic cell; GM-CSF, granulocyte-macrophage colony-stimulating factor; iBALT, inducible bronchus-associated lymphoid tissue; IFN, interferon; ILC, innate lymphoid cells; IM, interstitial macrophages; IL, interleukin; KLRG, killer-cell lectin like receptor G; MAIT, mucosal-associated invariant T-cells; PD, programmed cell death protein; sIgA, secretory immunoglobulin A; TCR, T-cell receptor; TNF, tumor necrosis factor; TRM, tissue-resident memory T-cell. **Created with** **BioRender.com.**

The study of human lung immunity gives the opportunity to interrogate the interaction between the host and *M.tb* bacilli at the site of natural infection. However, significant barriers exist. Obtaining high quality respiratory samples for immunological analysis generally requires invasive sampling (see Figure 1). The scarcity of these resources in areas with the highest burdens of TB disease, coupled with the costs and ethical considerations of invasive sampling, may explain the relative lack of immunological studies focussing on the human pulmonary microenvironment (4).

How best to sample the human pulmonary compartment remains the subject of debate. Sputum (induced or spontaneous) is often too contaminated, for example with upper airway epithelial cells and microbes, to provide detailed immunological analysis of the lower respiratory tract. Bronchoalveolar lavage (BAL) can be used to obtain bronchoalveolar cells (66). Studies comparing BAL cells to lung tissue biopsies in healthy controls and TB patients suggest that BAL cells are a reasonable representation of lung cellular composition (67). However, this assumption may not hold true for HIV infected individuals, where significant depletion of lung interstial CD4^+^ T cells may occur despite relatively normal CD4^+^ T cells levels in bronchoalveolar cells (68).

Where comparative data does exist, it suggest there is a significant difference in immunological activity and therefore possible biomarkers of disease and protection in the lungs compared to the peripheral blood. Schwander et al. found compartmentalized markers of active TB disease, with significantly increased levels of activated T lymphocytes (CD69+ HLA DR +) seen in bronchoalveolar cells of patients with active TB compared to healthy controls, whereas in PBMCs there was no difference across groups (69). PBMC in TB patients are hyporesponsive, with respect to both frequency of IFN-γ producing cells and DNA synthesis, to both mycobacterial and non-mycobacterial antigens compared to healthy subjects. Conversely, bronchoalveolar cells from affected lung segments in TB patients show increased responses to mycobacterial antigens, suggesting significant localization of antigen–specific cells within the affected lungs during active pulmonary TB (70).

Pulmonary TB disease is characterized by an enhancement of local Th1-mediated immunity, with increased IL-12 and IFN-γ production within affected lung segments (70). Despite this apparently functional local Th1-mediated immune response, there is clearly failure to control *M.tb* in those with active disease. Suppressive cytokines, including IL-4, TGF-β and IL-10, are increased in bronchoalveolar cell samples of active TB compared with healthy controls (71) and may represent distinct local immunosuppressive mechanisms that interfere with Th1-mediated effectors in the bronchoalveolar environment. One difficulty in studying the respiratory mucosal immune response to *M.tb* infection in humans is the inability to define precisely the time of infection. Due to the varying clinical course and potential for latency, active disease may only be diagnosed months or years after the point of infection. In other diseases, such as influenza, malaria and typhoid, controlled human infection models (CHIMs) have been used to interrogate the immune response and can also be used to evaluate vaccine efficacy. Treatment for active TB disease requires a lengthy combination of potentially toxic medications, and proof of definitive cure may not be possible. For these reasons, a CHIM with *M.tb* would not be ethical. However, use of alternative mycobacterial models to mimic *M.tb* infection are being explored. For example, BCG may be used as a surrogate, as it does not cause active disease in immunocompetent humans but is a live replicating mycobacteria that stimulates an immune response. Interrogation of the pulmonary mucosal immune response following a defined time point infection with BCG may lead to greater understanding of key immunological mechanisms, in particular in the early stages of infection.

Bronchoscopic instillation of BCG into lung segments of healthy, HIV-negative participants in South Africa with a range of TB phenotypes was shown to be safe and resulted in changes to differentially expressed genes and proteomics in the BAL fluid which were not detectable in the blood, suggesting a highly localized response (72). Studies in our group are ongoing to define the human innate and adaptive immune response to a defined time point challenge with aerosol BCG, and specifically comparing the peripheral and pulmonary compartment (Clinical trials.gov/NCT03912207). Parallel ongoing studies in non-human primates will add value to this work.

## Discussion

Growing evidence shows that immunological responses are compartmentalized and biomarkers present in the peripheral blood may be poorly representative of important, local effects within the lungs. Innate, trained and adaptive components of the pulmonary immune system are likely to play an interconnected role in protection, with distinct features of lung mucosal immunity such as alveolar macrophages, BALT and T_RM_ all warranting further investigation. The characterization of these immunological responses at the natural site of *M.tb* infection is of paramount importance, both in to increase our understanding of pathogenesis and more specifically to aid rational vaccine development.

Pre-clinical animal models play a key role in defining the pulmonary immune response to both *M.tb* and systemic and mucosally-delivered TB vaccines. Carefully designed small studies in humans can complement and add to these pre-clinical studies. Interrogating the initial stages of *M.tb* immunity in human lungs, for example in healthy household contacts, would have the potential to distinguish biomarkers of protective immunity (COP) at the site of initial host contact with *M.tb*. Logistical and ethical difficulties in obtaining invasive human pulmonary sample in these circumstances mean that more novel investigative strategies may be needed.

Vaccine development in TB faces a paradox—a vaccine-induced COP can only be validated in large field trials of an effective vaccine. However, selection of which candidate vaccines to take forward for such costly trials requires some level of discrimination. As evidenced by the compartmentalized nature of immunological biomarkers in both human and animal models, peripheral blood biomarkers, whilst easier to obtain, may not be the best choice of read-out for rational vaccine selection.

CHIMs in healthy volunteers, either with BCG or potentially in future with rationally attenuated *M.tb* strains, may prove an alternative strategy to delineate human immunological response to a defined time point infection (73). Davids et al. human lung challenge model showed that responses to *in-vitro* and *in-vivo* PPD and BCG stimulation were significantly different, raising the prospect that the study of vaccine-induced immune biomarkers of protection may need to focus more on lung mycobacterial challenges and sampling, rather than peripheral blood (72). Given the considerable technical obstacles this approach faces, it could prove more likely that if pulmonary correlates of protection (or disease) are identified, systemic surrogate markers may be identifiable that can then be more easily appraised in future studies.

## Author Contributions

HMo wrote the first draft of the manuscript. HMo and HMc contributed to manuscript revision and read and approved the submitted version.

## Conflict of Interest

The authors declare that the research was conducted in the absence of any commercial or financial relationships that could be construed as a potential conflict of interest.
